# Draft Genomes Sequences of 11 *Geodermatophilaceae* Strains Isolated from Building Stones from New England and Indian Stone Ruins found at historic sites in Tamil Nadu, India

**DOI:** 10.7150/jgen.76121

**Published:** 2022-09-21

**Authors:** Nathaniel J. Ennis, Dhanasekaran Dharumadurai, Joseph L. Sevigny, Ryan Wilmot, Sulaiman M. Alnaimat, Julia G. Bryce, W. Kelley Thomas, Louis S. Tisa

**Affiliations:** 1 Department of Molecular, Cellular, and Biomedical Sciences, University of New Hampshire, Durham, New Hampshire, USA.; 2 Departments of Microbiology, Bharathidasan University, Tiruchirappalli, Tamil Nadu, India.; 3 Hubbard Center for Genome Studies, University of New Hampshire, Durham, New Hampshire, USA.; 4 Department of Earth Sciences, University of New Hampshire, Durham, NH, USA.; 5 Present address: Seres Therapeutics, Cambridge, MA, USA.; 6 Present address: Department of Medical Analysis, Al-Hussein Bin Talal University, Ma'an, Jordan.

**Keywords:** Genomes, Stones, Ruins, Climate, Geochemistry, Geodermatophilaceae, Actinobacteria.

## Abstract

Metagenomic analysis of stone microbiome from samples collected in New England, USA and Tamil Nadu, India identified numerous Actinobacteria including *Geodermatphilaceae*. A culture-dependent approach was performed as a companion study with this culture-independent metagenomic analysis of these stone samples and resulted in the isolation of eleven *Geodermatphilaceae* strains (2 *Geodermatophilus* and 9 *Blastococcus* strains). The genomes of the 11 *Geodermatphilaceae* strains were sequenced and analyzed. The genomes for the two *Geodermatophilus* isolates, DF1-2 and TF2-6, were 4.45 and 4.75 Mb, respectively, while the Blastococcus genomes ranged in size from 3.98 to 5.48 Mb. Phylogenetic analysis, digital DNA:DNA hybridization (dDDH), and comparisons of the average nucleotide identities (ANI) suggest the isolates represent novel *Geodermatophilus* and *Blastococcus* species. Functional analysis of the *Geodermatphilaceae* genomes provides insight on the stone microbiome niche.

## Introduction

Stone surfaces provide a harsh environment with limited nutrient and water availability, exposure to lethal UV irradiation, potential contact with toxic metals and metalloids, and cycles in temperature variation 1-4. Despite these seemly inhospitable conditions, stone surfaces can support microbial life and well-defined communities. Because of their hyphal nature, Actinobacteria have been considered a primary colonizer of rock that then helps promote the growth of successive microbial colonizers. Members of the family *Geodermatophilaceae* have also been consistently isolated from stone surfaces and interiors 5.

We have been investigating the stone microbiome across a variety of lithologies three sites (North Africa, Southern Tamil Nadu, India and New England, USA) using culture-independent metagenomic approaches 3, 6, 7. To supplement this metagenomic approach, a culture-dependent approach was taken to isolate Actinobacteria from two of these sites (Southern Tamil Nadu, India and New England, USA). This study focuses on the genomes of *Blastococcus* and *Geodermatophilus*, two genera of the family *Geodermatophilaceae,* of bacterial strains that were isolated from samples obtained at these sites.

## Material and Methods

### Stone samples

Stone samples were obtained from historic sites in Tamil Nadu, India and at three different colonial sites in New England 6, 7. These stone samples were used in culture-independent studies to determine the stone microbiome structure 6, 7. These samples were also used to obtain bacterial isolates for culture-dependent studies.

### Isolation of Bacteria Associated with Stone Surfaces

Stone samples were crushed aseptically with a surface-sterilized rock hammer in a Biosafety hood. Crushed rock samples were reduced to a powder by grinding with a sterile mortar and pestle. The pulverized stone samples were used to isolate stone-dwelling bacteria. Table 1 shows the stone samples and other pertinent information on the 11 *Geodermatophilaceae* isolates used in this study. For this approach, pulverized stone (0.5g) was suspended in 5 mL of sterile phosphate-buffered saline (PBS) solution 8 and mixed thoroughly on a vortex mixer for 1 min. Stone suspensions were serially diluted in PBS from 10^-1^ to 10^-6^ dilutions. For each stone sample, 100 uL of the 10^-4^, 10^-5^, and 10^-6^ dilutions were spread plated onto the following media types: Czapek supplemented with yeast extract (DSMZ medium 130; 9), Luedemann agar (DSMZ medium 877; 10), R2A agar (DSMZ medium 830; 11), and Starch Casein agar 12. Cycloheximide (50 ug/mL final concentration) was added to the growth media to inhibit fungal growth. These growth media were chosen to select for Actinobacteria or other slow-growing bacteria 9, 11. The plates were sealed with parafilm to retain moisture and were incubated at 28^o^C for two months before attempting to isolate individual colonies. Colonies were chosen for isolation based primarily on pigmentation indicating UV tolerance, but also based on distinct colony morphology and slow growth rate (one week or more of incubation needed for colony growth). Individual colonies were purified on the same medium that isolation was accomplished. All purified isolates were grown for three to five days in their appropriate medium and prepared for long-term storage at -80^o^C by mixing the culture with an equal volume of 60% glycerol. Among the two sampling regions, a total of 85 bacterial isolates were purified, identified, and stored.

### Extraction of Genomic DNA from Bacterial Isolates

Isolates were grown for three to five days in Czapek broth supplemented with yeast extract. Genomic DNA (gDNA) of the bacterial stone isolates was extracted by the cetyl trimethylammonium bromide (CTAB) method 13. The extracted gDNA was suspended in Tris-EDTA (TE) buffer and treated with RNase to remove RNA. The extracted DNA was quantified using a Nanodrop 2000c (Thermo Fisher Scientific, Waltham, MA).

### Amplification of Bacterial Isolate 16S rRNA Genes

To identify the isolated stone-dwelling bacteria, the 16S rRNA gene of each isolate was amplified through PCR using the extracted gDNA of each isolate. The gDNA was combined with OneTaq Hot Start Polymerase (New England Biolabs, Ipswich, MA) and primers A 7-26f (5'-CCG-TCG-ACG-AGC-TCA-GAG-TTT-GAT-CCT-GGC-TCA-3') and B 1523-1504r (5'-CCC-GGG-TAC-CAA-GCT-TAA-GGA-GGT-GAT-CCA-GCC-GCA-3'), as described previously 14. The conditions for thermal cycling were as follows: an initial denaturation step at 95^o^C for 5 min was followed by 35 cycles of denaturation at 95^o^C for 30 s, primer annealing at 55^o^C for 30 s, and extension at 68^o^C for 2 min, with the final cycle followed by a 10 min extension at 68^o^C. The amplified PCR products were purified using the QiaQuick PCR Purification Kit following the manufacturer's protocol (Qiagen, Hilden, Germany). The presence and approximate size of the 16S gene was verified through gel electrophoresis. Amplified PCR products were quantified using the Qubit Fluorometric Quantitation system (Thermo Fisher Scientific, Waltham, MA).

### Sanger Sequencing and Identification of Isolate 16S rRNA Genes

To obtain approximate identities of all stone-dwelling bacterial isolates, partial sequences corresponding to the mid-region of isolate 16S genes were obtained by Sanger Sequencing 15 through Genewiz according to the service guidelines (Genewiz Inc., South Plainfield, NJ) and using primer 907r (5'-CCG-TCA-ATT-CCT-TTR-AGT-TT-3'), as described previously 16. Partial sequences were aligned with the 16S ribosomal RNA sequence (Bacteria and Archaea) database using the Basic Local Alignment Search Tool (BLAST), through blastn Version 2.7.1 (NCBI, Bethesda, MD). Isolates were identified as the BLAST result with the highest alignment score.

The full 16S rRNA gene of isolates that were identified as being closely related to members of the Actinobacteria family *Geodermatophilaceae* was generated by Sanger sequencing as described above and by using additional sequencing primers to ensure coverage of the full 16S rRNA gene. The sequencing primers used were: A 7-26f, B 1523-1504r, C 704-685r (5'-TCT-GCG-CAT-TTC-ACC-GCT-AC-3') and D 1115-1100r (5'-AGG-GTT-GCG-CTC-GTT-G-3'), as described previously 14. The sequences for each of *Geodermatophilaceae* isolate were aligned to build a final consensus sequence of the full 16S rRNA gene using Serial Cloner Version 2.6.1 (Serial Basics, 2013). Full 16S rRNA gene sequences were aligned using BLAST as described above, and isolates were more accurately identified as the BLAST result with the highest alignment score.

Sequences of the full 16S rRNA genes of each Geodermatophilaceae isolate were submitted to GenBank 17 to add to the repository of publicly available DNA sequences and for future potential publication of novel isolates. GenBank accession numbers are MK239636-MK239646.

### Whole Genome Shotgun Sequencing of *Geodermatophilaceae* Isolates

To fully identify and explore the functional capacity of potentially novel *Geodermatophilaceae* isolates, whole genome shotgun sequencing was performed on the gDNA of the stone isolates identified as members of *Geodermatophilaceae* according to the 16S rRNA sequencing described above. Sequencing libraries for the eleven *Geodermatophilaceae* isolates were prepared using the Illumina Nextera Library Preparation protocol according to the manufacturer's instructions (Illumina Inc., San Diego, CA). Sequencing was completed on an Illumina HISeq 2500 HiSeq2500 platform (Illumina Inc., San Diego, CA) to produce 250 bp paired-end reads at the Hubbard Center for Genome Studies (UNH, Durham, NH). Raw sequencing data was demultiplexed using bcl2convert.

### Quality Filtering of Whole Genome Shotgun Sequencing Reads

Sequence data were trimmed using Trimmonatic version 0.36 18. Truseq adapters were trimmed with an allowance of two mismatches. Leading and trailing bases below quality of three were trimmed. The read was then scanned with a sliding window of 4 bps and trimmed if the average quality dropped below 30. Finally, reads were dropped if the length was less than 36 bps. Trimmed sequencing reads were assembled using SPAdes version 3.13 19 with default settings. The assembled genomes were annotated *via* the NCBI Prokaryotic Genome Annotation Pipeline (PGAP) 20. The assembly metrics and annotation features are given in Table 2. The identities of the strains were determined by a whole genome-based taxonomic analysis via the Type (Strain) Genome Server (TYGS) platform 21 (https://tygs.dsmz.de) including digital DNA:DNA hybridization (dDDH) values 22. Average nucleotide identity (ANI) analysis of these genomes was performed on the JSpeciesWS server (https://jspecies.ribohost.com/jspeciesws/) 23.

### Functional Assessment of *Geodermatophilaceae* Isolate Genomes

The genomes were analyzed for the Clusters of Orthrologous Groups (COG) functional categories to identify potential functionality of the isolates 24 by the use of the reCOGnizer tool workflow 25. Functional profiling of the *Geodermatophilaceae* isolate genomes was also performed using PALADIN (version 1.4.2) with the raw genomic reads 26. PALADIN detects open reading frames (ORFs) within the read data and converts them to protein sequences. Converted read protein sequences are aligned against a reference protein database using the Burrows-Wheeler Aligner 27. PALADIN then assigns protein functions to the aligned proteins detected within the genome based on the reference database. Here, the UniRef90 database was used as the reference protein database 28. Gene Ontology (GO) domains were assigned to each aligned genome protein sequence by parsing the UniProt report generated by PALADIN 24, 29. The three GO domains are cellular component, molecular function, and biological process, and were used to assign broad functional categories to the isolate genomes.

Due to the potential novelty of the *Geodermatophilaceae* isolates, the genomes were evaluated for the production of secondary metabolites that could aid in the survival on stone surfaces (*i.e.* carotenoids) or that could have biotechnology or medical applications (*i.e.* antibiotics). The assembled and filtered contigs of each genome were used to determine potential secondary metabolite production through the bacterial version of antiSMASH version 5.0 30.

## Results and Discussion

### Identification of Bacteria Isolated from Stone Surfaces

Several growth media were used to isolate a range of Actinobacteria, particularly members of the family Geodermatophilaceae, from stones. From the stones of the sampling regions, a total of 85 bacterial isolates were cultured, purified, and stored at -80^o^C.

A total of 40 bacteria were isolated from the stones collected from Tamil Nadu, India - 31 belonged to Actinobacteria (78%). Many of the isolated Actinobacteria belonged to the genera *Geodermatophilus*, *Blastococcus*, *Mycobacterium*, and *Micrococcus.* Nearly 90% of the Indian isolates were cultured from granite, while the rest were cultured from granodiorite. The 6 *Blastococcus* and 2 *Geodermatophilus* isolates were cultured from granite from several different sites (Table 1).

A total of 45 bacteria were isolated from New England stone samples - 25 belonged to Actinobacteria (56%). Prominent Actinobacteria cultured from New England stones included *Dermacoccus*, *Arthrobacter*, and *Blastococcus*. Other notable or unusual Actinobacteria included *Auraticoccus*, *Micromonospora*, and *Branchiibius*, among others. The *Blastococcus* isolates were cultured from the same built granite stone from Gay City, CT (Table 1).

### Sanger Sequencing of the Complete 16S rRNA Gene of *Geodermatophilaceae* Isolates

Of the 85 bacteria isolated from the sampled stones, 11 were identified as belonging to the family *Geodermatophilaceae*. The full 16S rRNA gene of the 11 *Geodermatophilaceae* isolates was determined. The consensus sequences of the *Geodermatophilaceae* isolate 16S rRNA genes, including the top BLAST result and percent identity to each result, are summarized in Table S1. Two isolates belonged to the genus *Geodermatophilus* and 9 belonged to the genus *Blastococcus*.

### Assembly of *Geodermatophilaceae* Isolate Genomes

The genomes of the 11 stone-dwelling isolates identified above as members of *Geodermatophilaceae* were shotgun sequenced. Assembly statistics and taxonomy assignments are summarized in Table 2. All isolate genomes were identified as belonging to the same genus as described by the full 16S rRNA gene sequence. Assembly lengths for *Geodermatophilus* genomes ranged from 4,451,532 to 4,725,362 base pairs, while the assembly lengths for *Blastococcus* genomes ranged in size from 3,927,160 to 5,476,194 base pairs. All genome assemblies were composed of less than 90 contigs, except for isolates DF01-2 and GayMR20, which contained 199 and 345 contigs, respectively. All genomes also had an N_50_ value of at least 30,000 base pairs. The average genome coverage was at least 230X for all genomes except for isolate GayMR20, which had approximately 80X average genome coverage. In addition, all isolates had a high G+C % value of 72% or higher, which is consistent with the high G+C % values found previously in *Geodermatophilaceae* isolates.

### Assessment of the Novelty of *Geodermatophilaceae* Stone Isolates

**Figure 1:**
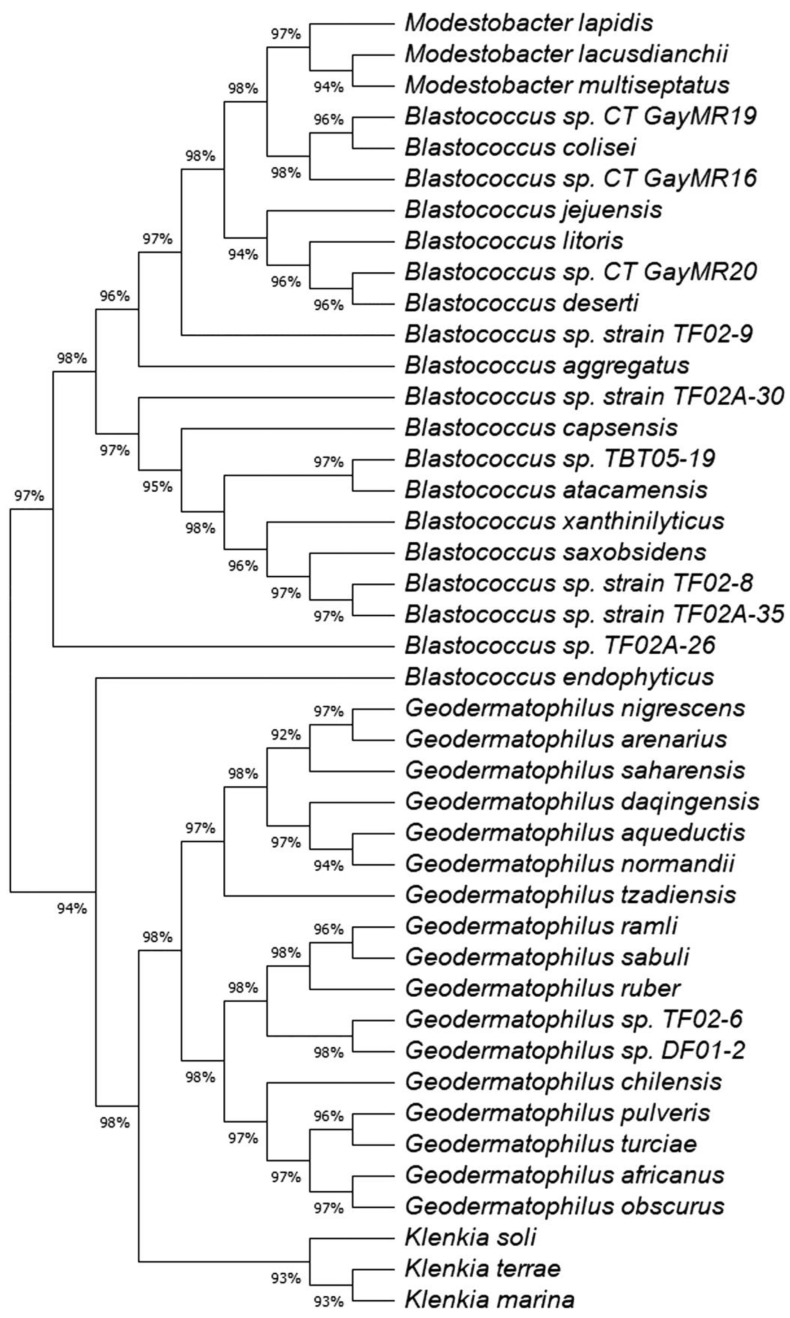
Maximum likelihood (ML) tree for the 16S rRNA sequences showing the position of the *Geodermatophilaceae* isolates. The tree consists of the following organisms and accession numbers in parenthesis *Blastococcus* sp. CT_GayMR20 (SPQM00000000); *Blastococcus* sp. CT_GayMR19 (SPQL00000000); *Blastococcus* sp. CT_GayMR16 (SPQK00000000); *Blastococcus* sp. TF02-9 (QOHH00000000); *Blastococcus* sp. TF02-8 MK239642; *Blastococcus* sp. TF02A-26 (QOHG00000000); *Blastococcus* sp. TF02A-30 (QOHJ00000000); *Blastococcus* sp. TF02A-35 (SPQP00000000);*Blastococcus* sp. TBT05-19 (QOHI00000000); *Geodermatophilus* sp. TF02-6 (QOHF00000000); *Geodermatophilus* sp. DF01-2 (SPQN00000000); *Geodermatophilus africanus* strain DSM 45422, isolate CF 11/1 (HE654550.1);*Geodermatophilus chilensis* strain B12T^T^ (KX943328.2); *Geodermatophilus normandii* DSM:45417, type strain CF 5/3^T^ (HE654546.1); *Geodermatophilus arenarius* type strain CF 5/4^T^ (HE654547.1); *Geodermatophilus daqingensis* strain WT-2-1 (KX881378.1); *Geodermatophilus tzadiensis* DSM45416, type strain CF5/2^T^ (HE654545.1);*Geodermatophilus ruber* DSM 45317, strain CPCC 201356 (EU438905); *Geodermatophilus sabuli* strain BMG 8133^T^ (LN626269.1);*Geodermatophilus aqueductis* BMG801^T^ DSM 46834 (LN626272); *Geodermatophilus obscurus* strain G20 DSM 43160 (CP001867); *Geodermatophilus amargosae* strain G96 DSM 46136 (HF679056; *Geodermatophilus saharensis* type strain CF5/5^T^ (HE654551); *Geodermatophilus dictyosporus,* type strain G-5^T^ (HF970584); *Geodermatophilus nigrescens* strain YIM 75980 (JN188947);*geodermatophilus pulveris* BMG825^T^ (LN626270; *Geodermatophilus poikilotrophus*, type strain DSM 44209^T^ (HF970583;*Geodermatophilus siccatus* strain DSM 45419, type strain CF6/1^T^ (HE654548);*Geodermatophilus marinus* strain LHW52908 (MG200147); *Klenkia marina*, strain YIM M13156 ^T^, DSM 45722 (LT746188); *Klenkia soli* strain PB34 16S^T^ (JN033772.1); *Klenkia terrae* strain PB261 (JN033773): *odestobacter lapidis* strain MON3.1^T^ (LN810544.1); *Modestobacter lacusdianchii* strain JXJ CY 19^T^ (KP986567.1); * Modestobacter multiseptatus* strain AA826T (Y18646.1); *Thalassiella azotivora* strain DSD2 (KT630890);*Nakamurella silvestris* strain S20-107 (KP899234;); *Blastococcus jejuensis* strain KST3-10 (DQ200983); *Blastococcus colisei* strain BMG 822^T^ (LN626273) ; *Blastococcus litoris* strain GP-S2-8^T^ (MH128378); *Blastococcus deserti* strain SYSU D8006 (MH553383); * Blastococcus aggregatus* strain DSM 4725^T^ (AJ430193.1); *Blastococcus endophyticus* strain YIM 68236^T^ (GQ494034); *Blastococcus capsensis* sp. BMG 804^T^ (LN626274); *Blastococcus xanthinilyticus* strain BMG 862^T^ (LN626275); *Blastococcus saxobsidens* type strain DSM 44509^T^ (FN600641); and *Blastococcus atacamensis* strain P6^T^ (KX926540). The evolutionary history was inferred by using the Maximum Likelihood method and Tamura-Nei model 34. The tree with the highest log likelihood (-7042.40) is shown. Initial tree(s) for the heuristic search were obtained automatically by applying Neighbor-Join and BioNJ algorithms to a matrix of pairwise distances estimated using the Tamura-Nei model, and then selecting the topology with superior log likelihood value. This analysis involved 47 nucleotide sequences. There were a total of 1570 positions in the final dataset. Evolutionary analyses were conducted in MEGA11 35.

**Figure 2:**
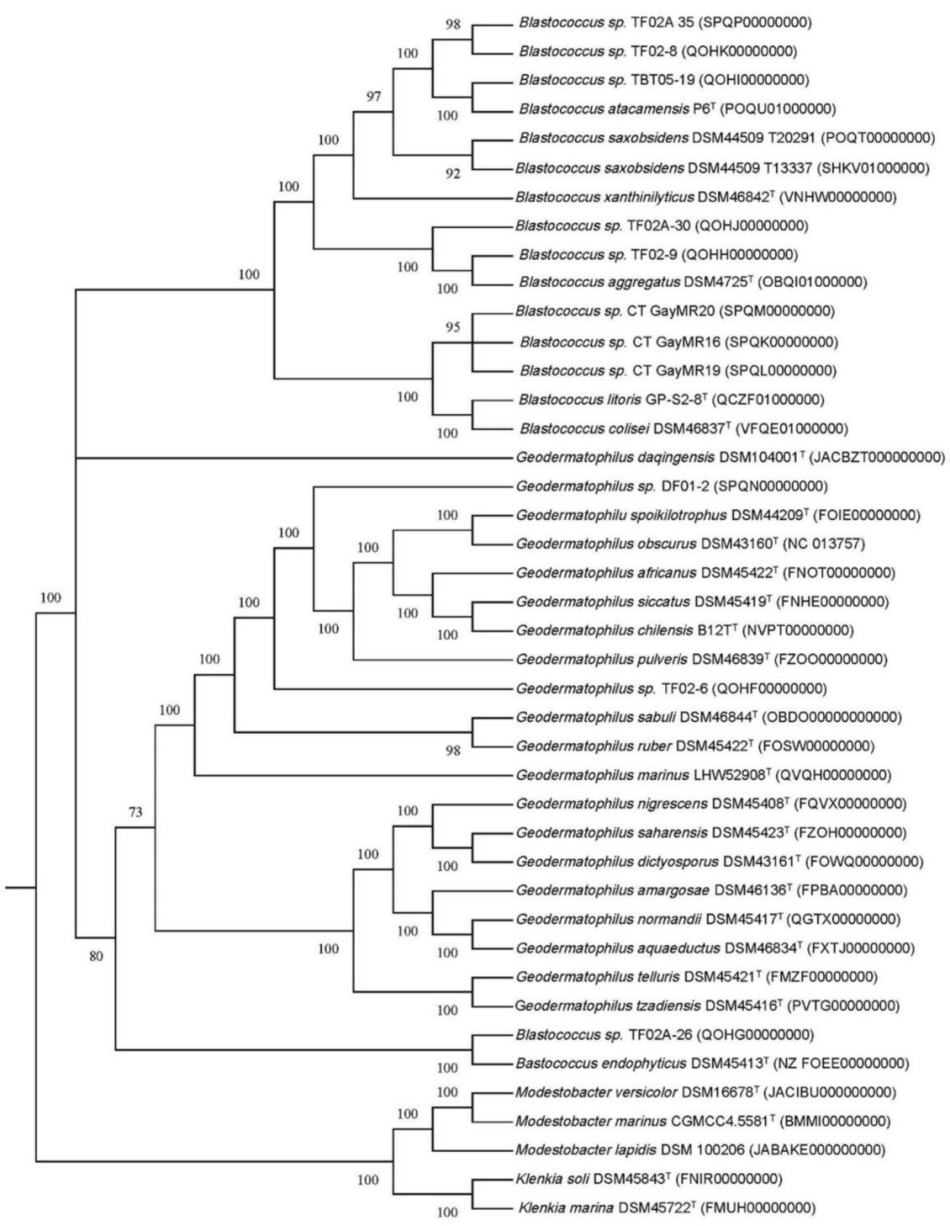
Tree inferred with FastME 2.1.6.1 36 from Genome BLAST Distance Phylogeny approach (GBDP) distances calculated from genome sequences. The branch lengths are scaled in terms of GBDP distance formula *d5*. The numbers above branches are GBDP pseudo-bootstrap support values > 60 % from 100 replications, with an average branch support of 95.1 %. The tree was rooted at the midpoint 37 and redrawn in MEGA11 35.

A maximum likelihood (ML) tree of the full 16S rRNA genes was constructed to determine the phylogeny of the 11 *Geodermatophilaceae* isolates (Fig. 1). Isolates DF01-2 and TF02-6 aligned near *G. ruber* and *G. sabuli*, but both were very distinct, indicating both as potential unique species. Similarly, all the *Blastococcus* isolates clustered several *Blastococcus* species, but were still distinct. Phylogenetic trees based on single genes are limited in scope. To obtain a better understanding of the phylogeny of the 11 isolates, a ML phylogenetic tree based on the entire genomes was constructed (Fig. 2). Phylogenetic analysis of the entire genomes confirmed 16 S rRNA gene phylogenetic tree and supports the idea that these isolates may represent potential novel species.

A whole genome-based taxonomic analysis via the Type (Strain) Genome Server (TYGS) platform 21 (https://tygs.dsmz.de) including digital DNA:DNA hybridization (dDDH) values 22 was performed to determine if these isolates represent new species (Fig. S1 and S2). The type-based species clustering using a 70% dDDH radius around each of the type strains was used as previously 31, while subspecies clustering was done using a 79% dDDH threshold as previously introduced 32. These data suggest that all *Blastococcus* and *Geodermatophilus* isolates are potential novel species. Average nucleotide identity (ANI) analysis of these genomes (Fig. S3 and S4) confirmed that idea with ANI values well below the threshold of 95% for species delineation 33.

### Functional Properties of *Geodermatophilaceae* Stone Isolates

Analysis of the 11 *Geodermatophilaceae* genomes for the number of genes associated with the Clusters of Orthrologous Groups (COG) functional categories showed that the pattern of distribution for each *Blastococcus* and *Geodermatophilus* isolate was like the patterns for *B. saxobsidens* DD2 and *G. obscurus* DSM 43160, respectively (Table S2 and S3).

**Figure 3:**
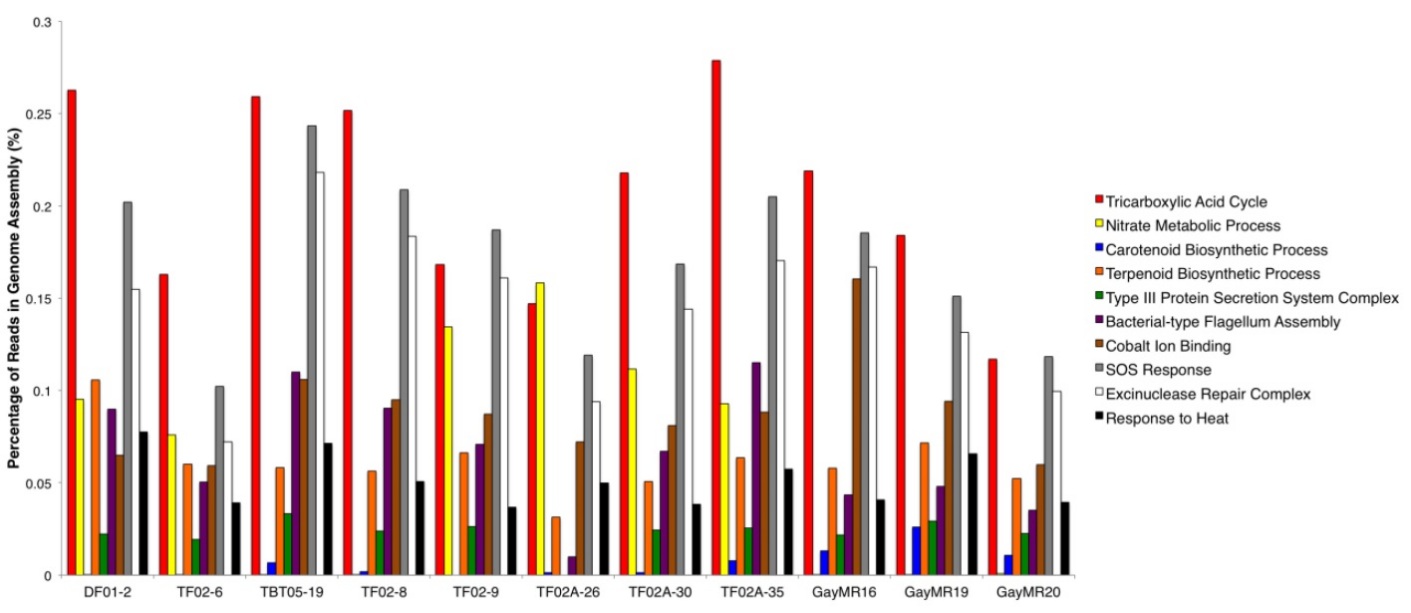
** Major Functions of *Geodermatophilaceae* Isolate Genomes.** The relative abundances of 10 major functions identified within the 11 *Geodermatophilaceae* isolate genomes are summarized. Function abundances are reported as the percentage of genomic reads mapped to each function within each genome.

To further determine the functional capacity of the *Geodermatophilaceae* stone isolates, the raw genomic reads were analyzed using PALADIN. A total of 2,691 GO Terms were identified within the 11 genomes - 910 belonged to the 'Biological Process' GO term type, 1,638 belonged to the 'Molecular Function' GO term type, and 143 belonged to the 'Cellular Component' GO term type. Figure 3 summarizes 10 major GO terms that were prominent within each isolate genome and were relevant to survival on stone surfaces. Among these 10 functions, three functions that were in high abundance within all 11 genomes were the Tricarboxylic Acid Cycle (GO:0006099), SOS Response (GO:0009432), and the Excinuclease Repair Complex (GO: 0009380). Other functions that were enriched but in lower abundance in all 11 genomes include the Terpenoid Biosynthesis Process (GO:0016114), Bacterial-type Flagellum Assembly (GO: 0044780), Cobalt Ion Binding (GO:0050897), and Response to Heat (GO:0009408). Interestingly, the Type III Protein Secretion System Complex (GO:0030257) was the most abundant secretion system type in these genomes and was found in all 11 isolates except for *Blastococcus* isolate TF02A-26. The Nitrate Metabolic Process (GO:0042126) was another broad metabolic function that was present in high abundance in most of the isolate genomes but was completely absent from *Blastococcus* isolates TBT05-19, TF02-8, GayMR16, GayMR19, and GayMR20. The Carotenoid Biosynthetic Process (GO:0016117) was present in surprisingly low abundance within the isolate genomes, despite the highly pigmented morphology of most members of *Geodermatophilaceae*. This function was present at very low abundance within both *Geodermatophilus* isolates (DF01-2 and TF02-6), *Blastococcus* isolates TF02-8, TF02A-26, and TF02A-30. This function was also completely absent within *Blastococcus* isolate TF02-9.

The antiSMASH version 5.0 program was also used on the assembled genomes of the 11 *Geodermatophilaceae* isolates to determine if the isolates had the potential to produce secondary metabolites, including antibiotics. The gene clusters detected in each isolate genome are summarized in Table 3. The Alkyl-O-Dihydrogeranyl-Methoxyhyrdoquinone biosynthesis gene cluster, under the Type 3 polyketide synthase (T3pks) metabolite type, was detected in every isolate genome. All isolate genomes also contained gene clusters associated with pigmentation production, in the forms of carotenoid or isorenierate biosynthesis. Many of these *Geodermatophilaceae* genomes also contained gene clusters associated with the production of antibacterial, antifungal, or even antiviral compounds, including stenothricin, pradimicin, nanchangmycin, istamycin, and fosfazinomycin. Interestingly, the Desferrioxamine B biosynthesis gene cluster, which is associated with siderophore iron-chelating activity, was detected in isolates TF02-8, TF02A-26, and TF02A-35. Several unknown secondary metabolites were also detected in isolates TF02-6, TF02-8, TF02-9, TF02A-30, TF02A-35, and GayMR20

In summary, we isolated 11 *Geodermatophilaceae* strains (9 *Blastococcus* and 2 *Geodermatophilus* isolates) and sequenced their genomes. These isolates represent potential novel species of these two bacterial genera. Analysis of their genomes revealed several unique traits that could play a role in their ecological niche.

**Data availability.** The draft genome sequences of these bacterial strains have been deposited in GenBank under the accession numbers listed in Table 2. Both the assembly and raw reads are available at DDBJ/ENA/GenBank under BioProject numbers: PRJNA478225, PRJNA478231 PRJNA478233, PRJNA478236, PRJNA478237, PRJNA478240, and PRJNA480027.

## Supplementary Material

Supplementary figures and tables.

## Figures and Tables

**Table 1:** *Geodermatophilaceae* isolates used in this study and information on the stone sample.

Isolate	Medium^1^	Specific site of collection	Location	Coordinate (DMS)	Stone type	Climate	Stone Condition	Approximate Stone Age (Years)
TF02-8	Czpek	Outside rock damage area	Fort Tiruchirappalli, Tamil Nadu, India	10^o^49'40” N 78^o^41'49” E	Granite	Tropical Wet and Dry	Built	1,000-1,500
TF02-6	Czpek	Outside rock damage area	Fort Tiruchirappalli, Tamil Nadu, India	10^o^49'40” N 78^o^41'49” E	Granite	Tropical Wet and Dry	Built	1,000-1,500
TF02-09	Czpek	Outside rock damage area	Fort Tiruchirappalli, Tamil Nadu, India	10^o^49'40” N 78^o^41'49” E	Granite	Tropical Wet and Dry	Built	1,000-1,500
TF02A-26	Czpek	Temple wall outside	Fort Tiruchirappalli, Tamil Nadu, India	10^o^49'40” N 78^o^41'49” E	Granite	Tropical Wet and Dry	Built	1,000-1,500
TF02A-30	Czpek	Temple wall outside	Fort Tiruchirappalli, Tamil Nadu, India	10^o^49'40” N 78^o^41'49” E	Granite	Tropical Wet and Dry	Built	1,000-1,500
TF02A-35	Czpek	Temple wall outside	Fort Tiruchirappalli, Tamil Nadu, India	10^o^49'40” N 78^o^41'49” E	Granite	Tropical Wet and Dry	Built	1,000-1,500
TBT05-19	Czpek	Temple wall outside damage area	Thanjavur Big Temple, Tamil Nadu, India	10^o^46'58” N 79^o^7'54” E	Granite	Tropical Wet and Dry	Built	1,000-1,500
DF01-2	Czapek	Temple wall outside	Fort Dindigul, Tamil Nadu, India	10^o^21'39” N 77^o^57'42” E	Granite	Tropical Wet and Dry	Built	250-500
CT_GayMR16	R2A	Mill site foundation	Gay City State Park Hebron, CT, USA	41^o^43'34” N 72^o^26'24” W	Granite	Humid Continental	Built	150-200
CT_GayMR19	LDM	Mill site foundation	Gay City State Park Hebron, CT, USA	41^o^43'34” N 72^o^26'24” W	Granite	Humid Continental	Built	150-200
CT_GayMR20	LDM	Mill site foundation	Gay City State Park Hebron, CT, USA	41^o^43'34” N 72^o^26'24” W	Granite	Humid Continental	Built	150-200

^1^ Czapak, Czapek-Dox medium ( DMSZ medium 130 9; R2A medium (DMSZ medium 830^11^); LDM, Luedemann Medium (DMSZ medium 877 10).

**Table 2:** Genome Statistics.

Bacterial species	Isolate	Genebank accession no.	Numbers of reads	No. of contigs	Avg coverage (X)	Genome assembly size (bp)	N_50_ contig size (kb)	No. of CDSs	G + C Content (%)	No. of rRNAs	No. of tRNAs
*Blastococcus* sp.	TF02-8	QOHK00000000	16,767,887	33	1026.0	3,982,980	380.9	3,814	75	8	47
*Blastococcus* sp.	TF02A-30	QOHJ00000000	15,562.207	38	922.0	4,129,003	466.8	4,008	74	6	48
*Blastococcus* sp.	TF02-09	QOHH00000000	9,367,969	37	558.0	4,132,992	297.8	3,953	73	11	47
*Blastococcus* sp.	TBT05-19	QOHI00000000	16,871,453	25	683.0	3,927,066	476.9	3,774	74	6	47
*Blastococcus* sp.	TF02A-26	QOHG00000000	12,236.063	54	627.0	4,678,378	217.9	4,561	74	6	47
*Blastococcus* sp.	TF02A-35	SPQP00000000	7,862,418	87	809.5	3,930,523	46.8	3,884	74	5	47
*Blastococcus* sp.	CT_GayMR16	SPQK00000000	5,162,206	47	157.2	4,520,567	136.3	4,472	73	8	47
*Blastococcus* sp.	CT_GayMR19	SPQL00000000	6,471,936	42	154.7	4,574,936	102.4	4,354	73	8	47
*Blastococcus* sp.	CT_GayMR20	SPQM00000000	1,759,527	345	37.1	5,475,077	37.1	5,501	73	7	56
*Geodermatophilus* sp	DF01-2	SPQN00000000	4,109,200	199	385.2	4,449,339	29.9	4,305	75	6	47
*Geodermatophilus* sp	TF02-6	QOHF00000000	12,613,686	53	639.0	4,725,362	162,9	4,448	75	7	49

**Table 3:** Biosynthetic gene clusters for natural products found in the genomes from *Geodermatophilacea.*

Bacterial species	Isolate	No. of Biosynthetic gene clusters ^1^	NRPS ^2^	PKS ^3^	Terpene	Siderophore	Betalactone	Bacteriocin	Lanthipeptide
*Blastococcus* sp.	TF02-8	4	1	1	1		1		
*Blastococcus* sp.	TF02A-30	3	1	1	1				
*Blastococcus* sp.	TF02-09	4	2	1	1				
*Blastococcus* sp.	TBT05-19	3		1	1			lassopeptide	
*Blastococcus* sp.	TF02A-26	1			1				
*Blastococcus* sp.	TF02A-35	5		1	1	1	butyrolactone	1	
*Blastococcus* sp.	CT_GayMR16	2		1	1				
*Blastococcus* sp.	CT_GayMR19	3	1	1	1				
*Blastococcus* sp.	CT_GayMR20	6	1	1	1		1	indole	1
*Blastococcus saxobsidens*	DD2	6	1	1	1		1	lassopeptide	
*Geodermatophilus* sp.	DF01-2	4	1	1	1			NPSR-terpene hopene	
*Geodermatophilus* sp.	TF02-6	5	1	2	1		1		
*Geodermatophilus obscurus*	DSM 43160	6	3	2	1				

^1^Biosynthetic gene clusters were identified by the use of the antiSMASH software. ^2^NRPS: Nonribosomal peptide synthase. ^3^PKS: polyketide synthase including Type I, II, III, Trans-AT, and other types
