# Supplementary material for: Draft Genomes Sequences of 11 Geodermatophilaceae Strains Isolated from Building Stones from New England and Indian Stone Ruins found at historic sites in Tamil Nadu, India
Source: J Genomics. 2022 Sep 21;10:69–77. doi: 10.7150/jgen.76121 (PMC9516006; doi:10.7150/jgen.76121)
Supplement: Supplementary file 1 — Supplementary figures and tables. [file jgenv10p0069s1.pdf]

Table S1. Geodermatophilaceae Isolates cultured from Sampled Stones. A total of 11 bacteria identified as members of the Actinobacteria family Geodermatophilaceae were isolated from various sampled stones. Isolates were identified based on the full consensus 16S rRNA sequence and BLAST results against the non-redundant database.

Sample ID	Length (bp)	Closest Identified Taxa	Percent Identity	E-Value	GenBank Accession Number
DF01-2	1,488	*Geodermatophilus obscurus* strain DSM 43160	98%	0.0	MK239636
TF02-6	1,442	*Geodermatophilus obscurus* strain DSM 43160	96%	0.0	MK239641
TF02-8	1,498	*Blastococcus saxobsidens* strain BC444	99%	0.0	MK239642
TF02-9	1,484	*Blastococcus aggregatus* strain DSM 4725	99%	0.0	MK239643
TBT05-19	1,479	*Blastococcus saxobsidens* strain BC444	99%	0.0	MK239640
TF02A-26	1,478	*Blastococcus saxobsidens* strain BC444	99%	0.0	MK239644
TF02A-30	1,454	*Blastococcus saxobsidens* strain BC444	99%	0.0	MK239645
TF02A-35	1,470	*Blastococcus saxobsidens* strain BC444	99%	0.0	MK239646
GayMR16	1,450	*Blastococcus colisei* strain BMG 822	98%	0.0	MK239637
GayMR19	1,443	*Blastococcus colisei* strain BMG 822	98%	0.0	MK239638
GayMR20	1,444	*Blastococcus saxobsidens* strain BC444	98%	0.0	MK239639

Table S2. *Blastococcus* CDS classified into Cluster of orthologous Genes (COG)

COG	Description	***B. saxobsidens*** **DD2**	**TF02-8**	**TF02A-30**	**TF02-9**	**TBT05-19**	**TF02A-26**	**TF02A-35**	**_ CT_GayMR16**	**CT_GayMR19**	**CT_GayMR20**
**<u>Cellular Processes</u>**											
**D**	Cell cycle control, cell division, chromosome partitioning	46 (1.02%)	34 (0.91%)	38 (0.97%)	42 (1.09%)	34 (0.92%)	45 (1.01%)	39 (1.03%)	47 (1.08%)	43 (1.01%)	47 (0.88%)
**M**	Cell wall/membrane/ envelope biogenesis	176 (3.90%)	186 (4.99%)	158 (4.02%)	173 (4.49%)	169 (4.57%)	181 (4.07%)	161 (4.26%)	160 (3.67%)	152 (3.59%)	210 (3.93%)
**N**	Cell motility	34 (0.75%)	31 0.83%)	24 (0.61%)	28 (0.73%)	33 (0.89%)	30 (0.68%)	31 (0.82%)	38 (0.87%)	33 (0.78%)	40 (0.75%)
**O**	Posttranslational modification, protein turnover, chaperones	120 (2.66%)	126 3.38%)	122 (3.10%)	120 (3.12%)	105 (2.84%)	112 (2.52%)	111 (2.94%)	129 (2.96%)	121 (2.86%)	172 (3.22%)
**T**	Signal transduction mechanisms	310 (6.86%)	262 7.03%)	298 (7.58%)	309 (8.02%)	301 (8.14%)	346 (7.79%)	273 (7.23%)	305 (6.99%)	336 (7.93%)	397 (7.44%)
**U**	Intracellular trafficking, secretion, and vesicular transport	31 (0.69%)	30 0.80%)	25 (0.64%)	24 (0.62%)	26 (0.70%)	28 (0.63%)	26 (0.69%)	31 (0.71%)	36 (0.85%)	33 (0.62%)
**V**	Defense mechanisms	103 (2.28%)	67 (1.80%)	74 (1.88%)	67 (1.74%)	63 (1.70%)	94 (2.12%)	79 (2.09%)	77 (1.76%)	72 (1.70%)	88 (1.65%)
**W**	Extracellular structures	10 (0.22%)	7 (0.19%)	3 (0.08%)	2 (0.05%)	7 (0.19%)	2 (0.05%)	6 (0.16%)	7 (0.16%)	9 (0.21%)	9 (0.17%)
**Y**	Nuclear structure	0	0	0	0	0	0	0	0	0	0
**Z**	Cytoskeleton	0	0	0	0	0	0	0	0	0	0
**X**	Mobilome: prophages, transposons	26 (0.58%)	7 (0.19%)	10 (0.25%)	19 (0.49%)	6 (0.16%)	25 (0.56%)	10 (0.26%)	8 (0.18%)	7 (0.17%)	11 (0.21%)
**<u>Information Storage and Processing</u>**											
**A**	RNA processing and modification	0	0	0	0	0	0	0	0	0	0
**B**	Chromatin structure and dynamics	0	0	0	0	0	0	0	0	0	0
**L**	Replication, recombination and repair	133 (2.94%)	112 (3.01%)	122 (3.10%)	114 (2.96%)	114 (3.08%)	117 (2.63%)	105 (2.78%)	125 (2.86%)	113 (2.67%)	143 (2.68%)
**K**	Transcription	295 (6.53%)	239 (6.41%)	283 (7.20%)	253 (6.57%)	234 (6.32%)	358 (8.06%)	255 (6.75%)	315 (7.22%)	301 (7.10%)	391 (7.32%)

**J**	Translation, ribosomal structure and biogenesis	199 (4.40%)	194 (5.21%)	195 (4.96%)	187 (4.85%)	201 (5.43%)	195 (4.39%)	204 (5.40%)	195 (4.47%)	193 (4.56%)	222 (4.16%)
**<u>Metabolism</u>**											
**C**	Energy production and conversion	241 (5.33%)	186 (4.99%)	199 (5.06%)	192 (4.98%)	168 (4.54%)	219 (4.93%)	169 (4.47%)	208 (4.77%)	235 (5.55%)	252 (4.75%)
**E**	Amino acid transport and metabolism	275 (6.09%)	215 (5.77%)	238 (6.05%)	237 (6.15%)	234 (6.32%)	231 (5.20%)	238 (6.30%)	238 (5.45%)	251 (5.91%)	303 (5.68%)
**F**	Nucleotide transport and metabolism	88 (1.95%)	85 (2.28%)	90 (2.29%)	82 (2.13%)	91 (2.46%)	99 (2.23%)	84 (2.22%)	93 (2.13%)	103 (2.43%)	110 (2.06%)
**G**	Carbohydrate transport and metabolism	158 (3.50%)	141 (3.78%)	168 (4.27%)	141 (3.66%)	136 (3.68%)	153 (3.44%)	151 (4.00%)	138 (3.16%)	183 (4.32%)	205 (3.84%)
**H**	Coenzyme transport and metabolism	235 (5.20%)	202 (5.42%)	205 (5.21%)	199 (5.17%)	200 (5.41%)	214 (4.82%)	200 (5.29%)	227 (5.20%)	218 (5.15%)	280 (5.25%)
**I**	Lipid transport and metabolism	251 (5.56%)	191 (5.12%)	201 (5.11%)	207 (5.37%)	200 (5.41%)	288 (6.48%)	201 (5.32%)	262 (6.00%)	219 (5.17%)	296 (5.55%)
**P**	Inorganic ion transport and metabolism	186 (4.12%)	151 (4.05%)	180 (4.58%)	154 (4.00%)	138 (3.73%)	175 (3.94%)	158 (4.18%)	160 (3.67%)	151 (3.56%)	178 (3.33%)
**Q**	Secondary metabolites biosynthesis, transport and catabolism	60 (1.33%)	52 (1.40%)	53 (1.35%)	47 (1.22%)	44 (1.19%)	69 (1.55%)	48 (1.27%)	57 (1.31%)	72 (1.70%)	79 (1.48%)
**<u>Poorly Characterized</u>**											
**S**	Function unknown	53 (1.17%)	58 (1.56%)	63 (1.60%)	59 (1.53%)	51 (1.38%)	60 (1.35%)	57 (1.51%)	67 (1.53%)	81 (1.91%)	74 (1.39%)
**R**	General function prediction only	297 (6.57%)	239 (6.41%)	247 (6.28%)	257 (6.67%	250 (6.76%)	286 (6.44%)	224 (5.93%)	302 (6.92%)	281 (6.63%)	354 (6.63%)
	Not in COGs	1191 (26.36%)	912 (24.47%)	934 (23.76%)	939 (24.38%)	894 (24.16%)	1115 (25.10%)	451 (11.94%)	1176 (26.94%)	1027 (24.24%)	1444 (27.05%)

Table S3. *Geodermatophilus* CDS classified into Cluster of orthologous Genes (COG)

COG	Description	*Geodermatophilus obscurus* DSM 43160	*Geodermatophilus* sp. DF01-2	*Geodermatophilus* sp. TF02-6
**<u>Cellular Processes</u>**				
**D**	Cell cycle control, cell division, chromosome partitioning	46 (0.94%)	41 (1.01%)	40 (0.94%)
**M**	Cell wall/membrane/envelope biogenesis	205 (4.19%)	152 (3.74%)	160 (3.77%)
**N**	Cell motility	35 (0.71%)	30 (0.74%)	36 (0.85%)
**O**	Posttranslational modification, protein turnover, chaperones	119 (2.43%)	108 (2.65%)	115 (2.71%)
**T**	Signal transduction mechanisms	342 (6.99%)	288 (7.08%)	256 (6.03%)
**U**	Intracellular trafficking, secretion, and vesicular transport	27 (0.55%)	27 (0.66%)	23 (0.54%)
**V**	Defense mechanisms	100 (2.04%)	93 (2.29%)	105 (2.47%)
**W**	Extracellular structures	7 (0.14%)	8 (0.20%)	4 (0.09%)
**Y**	Nuclear structure	0	0	0
**Z**	Cytoskeleton	0	0	0
**X**	Mobilome: prophages, transposons	36 (0.74%)	15 (0.37%)	18 (0.42%)
**<u>Information Storage and Processing</u>**				
**A**	RNA processing and modification	0	0	0
**B**	Chromatin structure and dynamics	0	0	0
**L**	Replication, recombination and repair	126 (2.57%)	112 (2.75%)	114 (2.69%)
**K**	Transcription	362 (7.39%)	256 (6.29%)	303 (7.14%)
**J**	Translation, ribosomal structure and biogenesis	205 (4.19%)	191 (4.69%)	195 (4.59%)
**<u>Metabolism</u>**				
**C**	Energy production and conversion	251 (5.13%)	230 (5.65%)	252 (5.94%)
**E**	Amino acid transport and metabolism	269 (5.49%)	241 (5.92%)	255 (6.01%)
**F**	Nucleotide transport and metabolism	89 (1.82%)	87 (2.14%)	82 (1.93%)
**G**	Carbohydrate transport and metabolism	213 (4.35%)	139 (3.42%)	194 (4.57%)
**H**	Coenzyme transport and metabolism	243 (4.96%)	215 (5.28%)	234 (5.51%)
**I**	Lipid transport and metabolism	227 (4.64%)	271 (6.66%)	277 (6.53%)

**P**	Inorganic ion transport and metabolism	149 (3.04%)	145 (3.56%)	131 (3.09%)
**Q**	Secondary metabolites biosynthesis, transport and catabolism	79 (1.61%)	55 (1.35%)	80 (1.89%)
**Poorly Characterized**				
**S**	Function unknown	71 (1.45%)	50 (1.23%)	64 (1.51%)
**R**	General function prediction only	360 (7.35%)	278 (6.83%)	285 (6.72%)
	Not in COGs	1335 (27.27%)	1037 (25.49%)	1021 (24.06%)

Fig S1. **Digital DNA:DNA hybridization (dDDH) values for *Blastococcus* strains**

***Blastococcus* strain**	**TF02A-30**	**TF02A-26**	**TF02A 35**	**TF02-9**	**TF02-8**	**TBT05-19**	**CT GayMR20**	**CT GayMR19**	**CT GayMR16**	**DSM 4725**	**P6**	**DSM 46837**	**DSM 45413**	**GP-S2-8**	**DSM 44509**	**DSM 46842T**
Blastococcus sp. TF02A-30	100	23.4	25.2	25.9	23.1	25	24	23.8	23.7	26	24.7	24.3	23.2	24.6	24.8	24.9
Blastococcus sp. TF02A-26	23.4	100	23.7	23.2	24	23	22.8	22.5	22.5	22.9	23	22.5	28.5	22.9	23.2	23.1
Blastococcus sp. TF02A 35	25.2	23.7	100	24.9	31.7	32.3	23.8	23.5	23.3	24.4	31.3	24	23.1	24.2	28.3	26.2
Blastococcus sp. TF02-9	25.9	23.2	24.9	100	30	24.4	23.9	23.5	23.5	29.8	24.4	24.1	22.9	24.4	24.4	24.3
Blastococcus sp. TF02-8	23.1	24	31.7	30	100	30	23.5	23.1	23.1	24.2	29.3	24	22.6	24	27	25.6
Blastococcus sp. TBT05-19	25	23	32.3	24.4	30	100	23.8	23.4	23.2	24.4	34.3	24.2	22.8	24.4	28.1	26
Blastococcus sp. CT GayMR20	24	22.8	23.8	23.9	23.5	23.8	100	25.1	25.6	24	23.8	25.7	22.5	25.3	24.2	24
Blastococcus sp. CT GayMR19	23.8	22.5	23.5	23.5	23.1	23.4	25.1	100	25	23.6	23.4	24.4	22.5	24.3	23.6	23.4
Blastococcus sp. CT GayMR16	23.7	22.5	23.3	23.5	23.1	23.2	25.6	25	100	23.7	23.3	24.9	22.4	24.5	23.5	23.6
Blastococcus aggregatus DSM 4725	26	22.9	24.4	29.8	24.2	24.4	24	23.6	23.7	100	24.2	24.1	22.8	24	24.7	25.8
Blastococcus atacamensis P6	24.7	23	31.3	24.4	29.3	34.3	23.8	23.4	23.3	24.2	100	24.1	22.7	24.1	26.1	24.5
Blastococcus colisei DSM 46837	24.3	22.5	24	24.1	24	24.2	25.7	24.4	24.9	24.1	24.1	100	22.5	27.4	24.5	24.5
Blastococcus endophyticus DSM 45413	23.2	28.5	23.1	22.9	22.6	22.8	22.5	22.5	22.4	22.8	22.7	22.5	100	22.6	22.8	22.9
Blastococcus litoris GP-S2-8	24.6	22.9	24.2	24.4	24	24.4	25.3	24.3	24.5	24	24.1	27.4	22.6	100	24.4	24.2
Blastococcus saxobsidens DSM 44509	24.8	24.8	28.3	24.4	27	28.1	24.2	23.6	23.5	24.7	26.1	24.5	22.8	24.4	100	26.5
Blastococcus xanthinilyticus DSM 46842T	24.9	24.9	26.2	24.3	25.6	26	24	23.4	23.6	25.8	24.5	24.5	22.9	24.2	26.5	100

Fig. S2. Digital DNA:DNA hybridization(dDDH) values for *Geodermatophilus* strains

Geodermatophilus strain	DF01-2	TF02-6	B12T	DSM 45419	DSM 44209	DSM 43160	DSM 45422	DSM 46839	DSM 46844	DSM 45317	DSM 45417T	DSM 45421	DSM 45416	DSM 45423	DSM 43161
Geodermatophilus sp. DF01 2	100	29.7	32.5	32.1	32.4	32.3	31.4	30.2	27.6	27.5	29.1	25.3	25.4	24.8	24.7
Geodermatophilus sp. TF02-6	29.7	100	29.1	28.9	28.5	28.5	28.3	27.9	27.7	27.5	24.9	25.5	25.4	25.1	24.7
Geodermatophilus chilensis B12T	32.5	29.1	100	42.4	37.8	37.4	38.7	31.6	27.4	27.1	25.3	25.7	25.8	25.3	25.2
Geodermatophilus siccatus DSM 45419	32.1	28.9	42.4	100	38.3	38.2	39.3	31.4	27.1	26.7	25	25.5	26.5	25.1	25
Geodermatophilus poikilotrophus DSM 44209	32.4	28.5	37.8	38.3	100	54.6	36.8	31.6	27	26.6	24.9	25.5	25.6	25.2	25
Geodermatophilus obscurus DSM 43160	32.3	28.5	37.4	38.2	54.6	100	36.3	31.5	27	26.8	24.9	25.5	25.5	25.2	25
Geodermatophilus africanus DSM 45422	31.4	28.3	38.7	39.3	36.8	36.3	100	31.1	26.7	26.4	25	25.3	25.4	24.9	24.9
Geodermatophilus pulveris DSM 46839	30.2	27.9	31.6	31.4	31.6	31.5	31.1	100	26	25.7	24.3	24.9	25	24.8	24.6
Geodermatophilus sabuli DSM 46844	27.6	27.7	27.4	27.1	27	27	26.7	26	100	27.3	24	24.3	24.4	24.3	24.1
Geodermatophilus ruber DSM 45317	27.5	27.5	27.1	26.7	26.6	26.8	26.4	25.7	27.3	100	25.1	24.5	24.5	24.4	24.5
Geodermatophilus normandii DSM 45417	29.1	24.9	25.3	25	24.9	24.9	25	24.3	24	25.1	100	29.6	29.8	29.9	29.6
Geodermatophilus telluris DSM 45421	25.3	25.5	25.7	25.5	25.5	25.5	25.3	24.9	24.3	24.5	29.6	100	44.1	29.8	44
Geodermatophilus tzadiensis DSM 45416	25.4	25.4	25.8	26.5	25.6	25.5	25.4	25	24.4	24.5	29.8	44.1	100	29.9	53
Geodermatophilus saharensis DSM 45423	24.8	25.1	25.3	25.1	25.2	25.2	24.9	24.8	24.3	24.4	29.9	29.8	29.9	100	29.8
Geodermatophilus dictyosporus DSM 43161	24.7	24.7	25.2	25	25	25	24.9	24.6	24.1	24.5	29.6	44	53	29.8	100

Fig. S3. Average Nucleotide Identify (ANI) values for *Blastococcus* strains

***Blastococcus* strain**	TF02-8	**TF02-9**	**TF02A-26**	**TF02A-30**	**TF02A-35**	**TBT05-19**	**CT_GayMR16**	**CT_GayMR19**	**CT_GayMR20**	**DSM4725**	**P6**	**DSM 44268**	**DSM46838**	**DSM 45413**	**DSM 44205**	**DSM 44270**	**GP-S2-8**	**DSM 44272**	**DD2**	**DSM 44509**	**DSM 46842T**	**DSM 46786**	**DSM 46790**	**URHD0036**
Blastococcus sp. TF02-8	100	83.314	82.3787	83.968	87.8312	87.0665	82.4238	82.2995	82.5664	83.4549	86.6164	83.0569	81.9127	82.9412	83.3398	82.5899	83.2371	82.6147	84.6802	85.2883	84.4052	84.5065	83.0669	82.0635
Blastococcus sp. TF02-9	83.312	100	82.4895	84.7275	83.9925	83.6472	82.7156	82.5793	82.9738	87.1564	83.4163	90.1516	83.1919	82.0305	87.2643	83.0688	83.4975	82.805	83.2107	83.5546	83.4627	83.3752	83.4192	82.1212
Blastococcus sp. TF02A-26	82.377	82.488	100	82.7267	82.9762	82.3863	81.6567	81.8098	81.8619	82.1848	82.1318	82.0117	81.9704	86.4782	82.2451	81.8523	82.283	81.8276	82.1161	82.3527	82.3444	82.2421	82.2177	88.4595
Blastococcus sp. TF02A-30	83.968	84.73	82.7233	100	82.9762	84.1456	82.8805	82.821	83.1876	84.8462	83.7459	84.6007	83.5649	82.3762	84.7371	83.227	83.7904	83.1151	83.8542	84.0673	84.0455	83.9687	83.5382	82.3874
Blastococcus sp. TF02A_35	87.827	83.993	82.9775	82.9775	100	88.3379	82.6842	82.7564	83.011	83.6979	87.78	83.6367	83.4166	82.2984	83.7245	83.1759	83.6577	82.988	84.8216	86.1251	84.9661	85.0964	83.5696	82.3786
Blastococcus sp. TBT05-19	87.065	83.649	82.3855	84.1452	88.334	100	82.5798	82.5526	82.9729	83.6256	89.0216	83.4792	83.3674	82.0683	83.7226	83.6256	83.5617	84.8483	84.6169	86.0662	84.8732	82.2112	83.5695	82.2103
Blastococcus sp. CT_GayMR16	82.423	82.716	81.6608	82.8795	82.6848	82.5795	100	83.7094	84.2801	82.7595	82.4598	82.4873	83.631	81.4981	82.683	83.5603	83.6271	83.7491	82.7131	82.5909	82.7269	82.4374	83.9724	81.5993
Blastococcus sp. CT_GayMR19	82.301	82.583	81.8096	82.8182	82.7547	82.55	83.7067	100	83.8777	82.6434	82.3737	82.4354	83.2058	81.6255	82.5943	83.2318	83.2908	83.5357	82.6565	82.5643	82.6471	82.456	83.6933	81.6866
Blastococcus sp. CT_GayMR20	82.498	82.903	81.8122	83.0911	82.8997	82.8975	84.1872	83.7924	100	82.9611	82.4902	82.5146	82.5901	83.8547	81.5079	82.8753	83.903	83.9682	82.9993	82.8509	82.8103	82.8103	84.5523	81.4987
Blastococcus aggregatus DSM 4725	83.454	87.155	82.1896	84.8447	83.6975	83.6247	82.7598	82.6452	83.0353	100	83.333	87.4397	87.4398	81.9786	87.6459	83.0443	83.294	82.8715	83.5872	83.7541	83.5268	83.5297	83.424	82.0247
Blastococcus atacamensis P6	86.615	83.418	82.1335	83.7434	87.7793	89.0216	82.4592	82.3759	82.5646	83.3335	100	83.2778	82.9807	81.8204	83.3561	82.9374	83.1999	82.6354	84.4404	85.6709	84.6245	84.4805	83.1412	81.9532
Blastococcus aurantiacus DSM 44268	83.061	90.155	82.0116	84.5975	83.6365	83.4773	82.487	82.4361	82.6015	87.4397	83.2765	100	83.1561	81.8049	87.6459	82.5886	83.1804	83.1829	83.0274	83.3864	83.2557	83.1829	83.217	81.9593
Blastococcus desertis DSM 46838	82.946	83.192	81.9704	83.5629	83.4191	83.3663	83.6295	83.2068	83.9374	83.3665	82.9796	83.1553	100	81.8101	83.3234	85.3795	85.2486	84.7596	83.5598	83.348	83.6383	83.194	85.7971	81.7424
Blastococcus endophyticus DSM 45413	81.92	82.031	86.4761	82.3746	82.3034	82.0662	81.497	81.6249	81.545	81.9817	81.8188	81.8001	81.8094	100	82.0267	81.6062	81.9413	81.5621	81.9865	82.0801	82.1658	82.0665	81.8397	86.0903
Blastococcus fimeti DSM 44205	83.339	87.265	82.2449	84.7358	83.724	83.7222	82.6824	82.5958	82.9244	87.646	83.3555	87.646	83.3235	82.0273	100	83.149	83.4014	82.8802	83.3432	83.6171	83.4919	83.4047	83.4091	82.1674
Blastococcus haudaquaticus DSM 44270	82.587	83.069	81.853	83.2256	83.1745	83.199	83.5585	83.233	83.9782	83.0399	82.9359	83.0239	85.379	81.6076	83.1488	100	85.7098	84.8818	83.0672	83.1851	83.037	82.9212	86.4194	81.5964
Blastococcus litoris GP-S2-8	83.24	83.499	82.2827	83.7968	83.6535	83.5617	83.6274	83.2974	84.2023	83.293	83.1999	83.1808	85.2496	81.9396	83.3957	85.7115	100	84.8704	83.2842	83.3647	83.3363	83.1861	86.9603	81.936
Blastococcus mobilis DSM 44272	82.608	82.804	81.827	83.1124	82.9877	84.8459	83.7468	83.5363	84.0822	82.8652	82.6334	82.5871	84.7606	81.5623	82.8797	84.8804	84.8674	100	83.0661	82.8468	82.9619	82.7591	85.2289	81.6006
Blastococcus saxobsidens DD2	84.682	83.188	82.1158	83.8522	84.8208	84.6177	82.7168	82.6623	83.0686	83.5949	84.438	83.0268	83.5423	81.991	83.343	83.067	83.284	83.0714	100	85.2071	86.5511	86.5511	83.3446	81.9554
Blastococcus saxobsidens DSM 44509	85.331	83.556	82.3535	84.0549	86.127	86.0743	82.5978	82.5664	82.8803	83.7506	85.6682	83.3893	83.3532	82.0801	83.6174	83.1857	83.3639	82.8441	85.2064	100	85.1869	84.8997	83.3917	82.1942
Blastococcus xanthinilyticus DSM 46842T	84.404	83.462	82.3398	84.0436	84.9651	84.8749	82.7261	82.6474	82.8809	83.5207	84.6216	83.2551	83.6342	82.162	83.4913	83.0376	83.3379	82.963	86.5519	85.1863	100	85.9831	83.3509	82.1552
Blastococcus sp. DSM 46786	84.506	83.375	82.2426	83.9761	85.0888	84.8459	82.4379	82.456	82.6205	83.5301	84.4787	83.1818	83.1922	82.0665	83.4044	82.9217	83.1922	82.7606	85.6132	84.8971	85.9837	100	83.2366	82.0393
Blastococcus sp. DSM 46790	83.065	83.419	82.2364	83.5361	83.5698	83.5682	83.9714	83.6937	84.583	83.424	83.14	83.2151	85.7985	81.8365	83.4094	86.4193	86.962	85.2263	83.3427	83.388	83.3449	83.2404	100	81.8585
Blastococcus sp. URHD0036	82.062	82.121	88.4557	82.3855	82.3776	82.2103	81.5979	81.6876	81.5616	82.0255	81.9513	81.9584	81.7474	86.0907	82.1666	81.5957	81.932	81.6012	81.9511	82.1942	82.1551	82.0434	81.8584	100

Fig. S4. Average Nucleotide Identify (ANI) values for *Geodermatophilus* strains.

	G. africanus DSM 45422 [T]	G. amargosae DSM 46136 [T]	G. aquaeductus DSM 46834 [T]	G. chilensis B12 [T]	G.s daqingensis DSM 104001 [T]	G.s nigrescens DSM 45408 [T]	G.s normandii DSM 45417 [T]	G.s obscurus DSM 43160 [T]	G.s poikilotrophus DSM 44209 [T]	G. pulveris DSM 46839 [T]	G. ruber DSM 45317 [T]	G. sabuli DSM 46844 [T]	G. saharensis DSM 45423 [T]	G.s siccatus DSM 45419 [T]	G. telluris DSM 45421 [T]	G. tzadiensis DSM 45416 [T]	Geodermatophilus sp. TF02-6	Geodermatophilus sp. DF01-2
Geodermatophilus africanus DSM 45422 [T]	100	80.76	80.73	88.31	79.12	80.64	80.75	87.99	88.03	85.02	81.59	82.10	80.92	88.92	81.24	81.34	82.77	84.98
Geodermatophilus amargosae DSM 46136 [T]	80.50	100	89.40	80.48	78.69	84.71	89.78	80.75	80.53	80.17	79.53	79.86	84.61	80.55	84.07	84.19	80.30	79.93
Geodermatophilus aquaeductus DSM 46834 [T]	80.62	89.95	100	80.82	79.11	85.19	91.15	80.62	80.82	80.25	79.99	80.26	84.82	80.83	84.68	84.60	80.46	80.44
Geodermatophilus chilensis B12 [T]	88.08	80.66	80.66	100	79.29	80.54	80.82	87.86	87.82	84.87	81.77	82.16	80.78	89.37	81.25	81.16	83.09	85.26
Geodermatophilus daqingensis DSM 104001 [T]	78.34	78.48	78.65	78.77	100	78.60	78.48	78.76	78.68	78.31	78.83	78.69	78.47	78.79	78.64	78.67	78.91	78.72
Geodermatophilus nigrescens DSM 45408 [T]	81.19	85.70	85.98	81.27	79.65	100	85.86	81.35	81.36	81.04	80.38	80.85	85.90	81.45	85.25	85.21	80.87	80.93
Geodermatophilus normandii DSM 45417 [T]	81.26	90.95	92.12	81.37	79.64	85.82	100	81.41	81.34	80.75	80.42	80.75	85.81	81.47	85.31	85.42	81.08	81.02
Geodermatophilus obscurus DSM 43160 [T]	87.92	80.83	80.65	87.96	79.47	80.52	80.83	100	92.90	85.28	81.78	82.32	81.00	88.75	81.36	81.40	83.13	85.46
Geodermatophilus poikilotrophus DSM 44209 [T]	88.42	81.09	81.10	88.40	79.71	81.01	81.17	93.41	100	85.63	81.96	82.66	81.38	89.37	81.87	81.83	83.46	85.88
Geodermatophilus pulveris DSM 46839 [T]	86.17	81.39	81.34	86.10	80.01	81.43	81.40	86.44	86.52	100	82.21	82.66	81.68	86.38	82.02	81.95	83.62	85.27
Geodermatophilus ruber DSM 45317 [T]	81.42	79.83	79.80	81.61	79.14	79.73	80.02	81.65	81.53	81.02	100	82.53	79.84	81.58	79.93	80.02	81.89	81.66
Geodermatophilus sabuli DSM 46844 [T]	81.42	79.69	79.77	81.87	78.81	79.63	79.70	81.75	81.75	81.00	82.23	100	79.85	81.84	79.93	80.03	82.03	81.76
Geodermatophilus saharensis DSM 45423 [T]	81.43	85.49	85.50	81.46	79.60	85.78	85.60	81.65	81.69	81.12	80.51	80.79	100	81.59	85.27	85.18	81.20	81.22
Geodermatophilus siccatus DSM 45419 [T]	88.90	81.03	81.07	89.62	79.37	80.93	81.10	88.73	89.04	85.27	81.85	82.53	81.12	100	81.69	81.73	83.44	85.48
Geodermatophilus telluris DSM 45421 [T]	81.50	85.06	85.18	81.66	79.47	85.01	84.99	81.62	81.96	81.33	80.45	80.75	85.25	81.90	*	91.21	81.32	80.99
Geodermatophilus tzadiensis DSM 45416 [T]	81.68	85.25	85.20	81.71	79.84	85.07	85.20	81.90	82.12	81.45	80.67	81.00	85.33	82.19	91.32	*	81.45	81.13
Geodermatophilus sp. TF02-6	82.84	80.67	80.76	83.35	79.62	80.38	80.63	83.17	83.28	82.68	82.47	82.84	80.83	83.62	81.21	81.03	100	83.58
Geodermatophilus sp. DF01-2	85.08	80.55	80.58	85.60	79.40	80.48	80.73	85.60	85.76	84.39	81.99	82.41	80.67	85.56	80.76	80.92	83.69	100
